# Single‐Catheter Versus Conventional Two‐Catheter Primary PCI in STEMI: A 1:1 Propensity Score‐Matched Cohort Study

**DOI:** 10.1155/cdr/4057540

**Published:** 2026-08-22

**Authors:** Xin Kou, Sen Lei, Hang Fu, Lingyun Gao

**Affiliations:** ^1^ Department of Cardiology, The First Affiliated Hospital of Chongqing Medical University, Chongqing, China, cqmu.edu.cn

**Keywords:** propensity score matching, single-catheter PCI, STEMI

## Abstract

**Background:**

Primary percutaneous coronary intervention (pPCI) is the gold‐standard reperfusion strategy for acute ST‐elevation myocardial infarction (STEMI). However, the conventional two‐catheter percutaneous coronary intervention (C‐PCI) workflow requires sequential use of diagnostic and guiding catheters, causing avoidable procedural delays. Single‐catheter percutaneous coronary intervention (SC‐PCI) uses a single universal guiding catheter for both coronary angiography and intervention, improving intraprocedural efficiency, but comparative data remain limited.

**Methods:**

This single‐center retrospective cohort study enrolled 268 consecutive STEMI patients undergoing transradial pPCI. Using 1:1 propensity score matching (PSM), baseline confounders were balanced between the SC‐PCI and C‐PCI groups. The primary endpoint was puncture‐to‐balloon (P2B) time; secondary endpoints included core procedural time metrics, intraprocedural exposure, safety, and short‐term outcomes. Exploratory subgroup analyses were performed to assess the consistency of treatment effect.

**Results:**

After PSM, 120 well‐balanced patients (60 per group) were included. SC‐PCI significantly reduced P2B time (median 9.5 vs. 15.5 min, *p* < 0.001), along with shorter puncture‐to‐angiography time, cath‐lab door‐to‐balloon time, fluoroscopy duration, and lower contrast volume (all *p* < 0.05). No statistically significant differences were observed in procedural success, overall complication rates, or in‐hospital mortality between groups. In exploratory subgroup analyses, the P2B benefit was consistent across subgroups, with no significant interactions (all *p* for interaction > 0.05).

**Conclusion:**

In this propensity‐matched STEMI cohort, SC‐PCI was associated with shorter P2B time and improved intraprocedural efficiency, with no significant differences in periprocedural safety outcomes, suggesting that SC‐PCI may be a promising strategy to optimize STEMI interventional workflows.

## 1. Introduction

Acute ST‐elevation myocardial infarction (STEMI) is a life‐threatening emergency that requires immediate myocardial reperfusion to limit ischemic injury and improve survival outcomes [1]. Primary percutaneous coronary intervention (pPCI) is recognized as the gold‐standard reperfusion strategy for STEMI, and advances over recent decades have effectively reduced system‐level delays, including prehospital diagnosis, emergency triage, and catheter laboratory activation [2, 3].

Despite these advances, procedure‐related delays within the catheter laboratory have become the major remaining barrier to further reducing total ischemic time. The conventional two‐catheter workflow requires separate diagnostic and guiding catheters: A diagnostic catheter is first used to identify the culprit lesion, followed by catheter exchange to introduce a dedicated guiding catheter for intervention [4, 5]. Such exchange inevitably prolongs time to reperfusion, increases vascular manipulation and complication risk, and raises both radiation exposure and contrast volume. The single‐catheter percutaneous coronary intervention (SC‐PCI) strategy overcomes these limitations by using one universal guiding catheter to complete both diagnostic angiography and therapeutic intervention without catheter exchange [4, 6]. This streamlined workflow can simplify the procedure, eliminate delays from catheter switching, reduce vascular manipulation, and potentially lower periprocedural complication rates and radiation exposure. Although recent studies have demonstrated the feasibility of the single‐catheter strategy in STEMI, robust evidence remains limited. Most studies focus solely on time‐to‐treatment endpoints, and data on subgroup consistency and generalizability are also scarce [7, 8].

Accordingly, the present study is aimed at systematically comparing procedural efficiency and safety of SC‐PCI versus conventional two‐catheter percutaneous coronary intervention (C‐PCI) in STEMI patients, with prespecified exploratory subgroup analyses to assess effect consistency across patient and lesion strata, and at providing additional real‐world evidence for optimizing emergency STEMI interventional care pathways.

## 2. Methods

### 2.1. Study Population and Grouping

**Figure 1:**
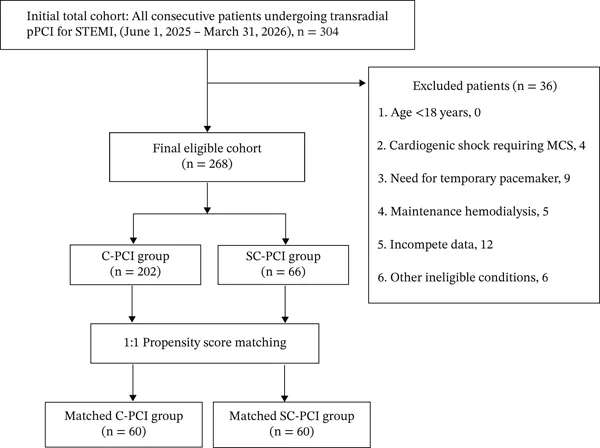
The study flow chart. Abbreviations: STEMI, ST‐segment elevation myocardial infarction; pPCI, primary percutaneous coronary intervention; C‐PCI, conventional two‐catheter percutaneous coronary intervention; SC‐PCI, single‐catheter percutaneous coronary intervention; MCS, mechanical circulatory support.

This was a single‐center, retrospective observational study based on our institutional cardiac catheterization laboratory (cath lab) database. Consecutive patients diagnosed with STEMI and undergoing transradial pPCI between June 1, 2025, and March 31, 2026, were initially screened. After applying the exclusion criteria, a total of 268 eligible patients were included. Among them, 66 patients were treated with the SC‐PCI strategy, whereas 202 received C‐PCI. To minimize baseline confounding between the two groups, 1:1 propensity score matching (PSM) was performed based on baseline clinical characteristics, resulting in a final matched cohort of 120 patients (60 in the SC‐PCI group and 60 in the C‐PCI group). Exclusion criteria were as follows: age < 18 years, cardiogenic shock requiring mechanical circulatory support, temporary pacemaker implantation, maintenance hemodialysis, incomplete data, and other ineligible conditions. All procedures complied with the ethical principles of the Declaration of Helsinki. The study protocol was approved by the local Institutional Review Board, with waiver of written informed consent granted for the retrospective, noninterventional design. The study flow chart was summarized in Figure 1.

### 2.2. The SC‐PCI Method and the C‐PCI Method

During the study period, both SC‐PCI and C‐PCI were available, with all patients treated per our standardized culprit‐first primary PCI protocol. Strategy selection was at operator discretion based on preference, experience, and catheter availability. All operators had at least 5 years of PCI experience, completed standardized SC‐PCI training, and were fully proficient in Ikari Left catheter manipulation for both coronary systems. For the SC‐PCI method, a 6‐ or 7‐Fr IL universal guiding catheter (Terumo Corporation, Tokyo, Japan) was utilized: After transradial puncture and sheath placement, a 0.035 ^″^ J Tip Teflon guidewire assisted advancing the IL catheter into the ascending aorta, routine back‐bleeding was performed to eliminate air embolism risk, nonculprit vessel angiography was conducted first (with the guidewire in the IL catheter′s secondary curve for right coronary artery [RCA] engagement and tertiary curve for left coronary artery [LCA] engagement) based on preoperative evaluations, followed by culprit vessel angiography, and PCI (including balloon dilatation and stent implantation) was performed directly without catheter exchange, with success defined as completing the entire procedure solely with the IL catheter. For the C‐PCI method, initial diagnostic angiography was performed using dedicated catheters (e.g., Judkins, Tiger; Terumo Corporation), and after confirming the culprit lesion, the diagnostic catheter was exchanged for a dedicated guiding catheter (type and size selected based on vascular anatomy and lesion characteristics, with the IL catheter also permitted), followed by PCI per standard techniques. Both groups used consistent biodegradable polymer‐coated everolimus‐eluting stents, antiplatelet therapy (aspirin + P2Y12 inhibitor loading), and anticoagulation (unfractionated heparin [UFH] to maintain an activated clotting time [ACT] > 250 s).

### 2.3. Endpoints

The primary endpoint was puncture‐to‐balloon (P2B) time, defined as the interval from successful radial arterial puncture and sheath insertion to the first balloon dilatation of the culprit infarct‐related artery.

Secondary endpoints included core procedural time metrics, intraprocedural exposure, safety and short‐term outcomes, specifically puncture‐to‐angiography (P2A) time, cath lab door‐to‐balloon (D2B) time, total procedural time (TPT), hospital D2B time, pre‐cath lab time, fluoroscopy time, contrast volume, total radiation dose, procedure success rate, device safety rate, overall complication rates, length of hospital stay, and in‐hospital mortality. Detailed definitions of all endpoints are provided in Appendix S1.

### 2.4. Statistical Analysis

Continuous variables were expressed as either the mean (standard deviation [SD]) or median (interquartile range [IQR]), depending on the normality and distribution of the data, as assessed using the Shapiro–Wilk test. Group comparisons employed parametric (Student′s *t*‐test) or nonparametric (Mann–Whitney *U*) tests, as appropriate. Categorical variables were represented as percentages and analyzed using the chi‐square test or Fisher′s exact test.

A 1:1 PSM analysis was performed to adjust for baseline imbalances between the SC‐PCI and C‐PCI cohorts. Propensity scores were estimated via a multivariable logistic regression model incorporating age, sex, body mass index (kg/m^2^), hypertension, diabetes mellitus, hyperlipidemia, smoking history, alcohol use, prior myocardial infarction, creatinine (*μ*mol/L), pro–B‐type natriuretic peptide (pg/mL), cardiac troponin I (ng/mL), left ventricular ejection fraction (%), and admission Killip classification. Patients were randomly ordered, and each SC‐PCI patient was 1:1 matched to the C‐PCI patient with the nearest propensity score using an optimized 0.2 caliper width, which achieved the best balance between intergroup homogeneity and sample size retention.

Prespecified exploratory subgroup analyses were performed to assess the consistency of P2B benefit across clinically relevant subgroups and explore potential effect heterogeneity. Patients were stratified into six subgroups: age (< 65 vs. ≥ 65 years, cutoff defined by the 2023 European Society of Cardiology [ESC] STEMI Guidelines), culprit vessel (left anterior descending [LAD], left circumflex [LCX], and RCA), vessel involvement (single‐vessel disease vs. multivessel disease, defined as ≥ 50% diameter stenosis in major epicardial arteries), lesion location (proximal, mid, and distal segments of the culprit vessel), number of stents (single vs. multiple stent implantation) and study periods (June–September 2025, October–December 2025, and January–March 2026) [9–11]. Within each subgroup, P2B time was compared between SC‐PCI and C‐PCI groups using Mann–Whitney *U* tests. Mean differences (MD) with 95% confidence intervals (CIs) and interaction tests were estimated using linear regression models, which were appropriate given the moderate sample size and the central limit theorem. All subgroup analyses were exploratory in nature and no adjustment for multiple comparisons was performed. Results were visualized using forest plots. A two‐sided *p* value < 0.05 was considered statistically significant. All analyses were performed with IBM SPSS Statistics Version 27.0 (IBM Corp., Armonk, New York, United States).

## 3. Result

### 3.1. Baseline Clinical Characteristics of the Unmatched and Matched Cohorts

In the prematched cohort, significant imbalances were observed between the SC‐PCI and C‐PCI groups for multiple key covariates, including alcohol use, hypertension, renal insufficiency, pro‐BNP level, and LVEF. Following 1:1 PSM, all baseline characteristics were well‐balanced between the two groups (all *p* > 0.05), which ensured the reliability of subsequent comparative analyses between groups (Table 1).

**Table 1:** Baseline clinical characteristics of the unmatched and matched cohorts.

Variables	Unmatched cohorts			Propensity score‐matched cohorts			
	SC‐PCI (*n* = 66)	C‐PCI (*n* = 202)	*p*	SC‐PCI (*n* = 60)	C‐PCI (*n* = 60)	*p*	
Age (year)	62.9 ± 13.9	63.6 ± 13.7	0.752	62.56 ± 14.5	64.6 ± 13.6	0.423	
Male, *n* (%)	57 (86.4)	157 (77.7)	0.129	52 (86.7)	49 (41.7)	0.453	
BMI (kg/m^2^)	24.6 ± 3.1	24.6 ± 3.8	0.982	24.3 ± 3.1	23.9 ± 3.5	0.447	
Tobacco use, *n* (%)	38 (57.6)	100 (49.5)	0.255	34 (56.7)	33 (55.0)	0.854	
Alcohol use, *n* (%)	30 (45.5)	57 (28.2)	0.009	24 (40.0)	26 (43.3)	0.711	
Hypertension, *n* (%)	46 (69.7)	106 (52.5)	0.014	38 (63.3)	36 (60.0)	0.707	
Diabetes mellitus, *n* (%)	29 (43.9)	84 (41.6)	0.737	26 (43.3)	28 (46.7)	0.714	
Hyperlipidemia, *n* (%)	27 (40.9)	63 (31.2)	0.147	23 (38.3)	24 (40.0)	0.852	
Renal insufficiency, *n* (%)	11 (16.7)	16 (7.9)	0.040	7 (11.7)	9 (15.0)	0.591	
Prior AMI, *n* (%)	3 (4.5)	12 (5.9)	0.669	3 (5.0)	1 (1.7)	0.309	
Killip class, *n* (%)			0.581			0.382	
I	56 (84.8)	155 (76.7)			51 (85.0)	47 (78.4)	
II	5 (7.6)	23 (11.4)			4 (6.7)	9 (15.0)	
III	1 (1.5)	5 (2.5)			1 (1.7)	2 (3.3)	
IV	4 (6.1)	19 (9.4)			4 (6.6)	2 (3.3)	
Creatinine (*μ*mol/L)	74.0 (61.8–87.7)	75.0 (62.0–87.0)	0.489		73.0 (61.0–86.8)	74.0 (60.0–85.0)	0.524
Pro‐BNP (pg/mL)	185.0 (109.2–1031.7)	452.0 (149.0–1740.0)	0.021		185.0 (107.8–1137.0)	444.5 (185.5–1202.6)	0.079
Troponin I (ng/mL)	3.6 (1.2–12.2)	6.0 (2.2–15.1)	0.058		4.3 (1.5–13.7)	7.1 (2.1–19.7)	0.153
LVEF (%)	53.4 ± 9.7	50.7 ± 8.8	0.045		52.9 ± 10.1	51.8 ± 9.1	0.557

*Note:* Values are median (IQR) or mean (± standard deviation) and number (percentage).

Abbreviations: AMI, acute myocardial infarction; BMI, body mass index; BNP, B‐type natriuretic peptide; C‐PCI, conventional percutaneous coronary intervention; LVEF, left ventricular ejection fraction; SC‐PCI, single‐catheter percutaneous coronary intervention.

### 3.2. Angiographic and Intraprocedural Characteristics

There were no significant intergroup differences in baseline angiographic and procedural characteristics, including culprit vessel distribution, extent of coronary vessel involvement, lesion location, preprocedural TIMI flow grade, rate of thrombus aspiration, number of implanted stents, and stent diameter (all *p* > 0.05). The total length of implanted drug‐eluting stents (DES) was significantly shorter in the SC‐PCI group than in the C‐PCI group (24.4 ± 5.5 vs. 27.0 ± 6.4 mm, *p* = 0.017). Postprocedural angiographic outcomes were comparable between the two cohorts, with 98.3% of patients in the SC‐PCI group and 100.0% in the C‐PCI group achieving final TIMI 3 flow (Table 2).

**Table 2:** Angiographic and interventional characteristics between SC‐PCI and C‐PCI groups.

Variables	SC‐PCI (*n* = 60)	C‐PCI (*n* = 60)	*p*
Culprit vessel, *n* (%)			0.151
LAD	22 (36.7)	30 (50.0)	
LCX	8 (13.3)	11 (18.3)	
RCA	29 (48.3)	19 (31.7)	
LM	1 (1.7)	0 (0.0)	
Vessel involvement, *n* (%)			0.597
1‐vessel disease	24 (40.0)	27 (45.5)	
2‐vessel disease	20 (33.3)	22 (36.7)	
3‐vessel disease	16 (26.7)	12 (18.3)	
Lesion location, *n* (%)			0.339
Proximal	29 (48.3)	33 (55.0)	
Mid	18 (30.0)	20 (33.3)	
Distal	13 (21.7)	7 (11.7)	
Pre‐TIMI flow grade, *n* (%)			0.657
TIMI 0	30 (50.0)	25 (41.7)	
TIMI 1	23 (38.3)	27 (45.0)	
TIMI 2	7 (11.7)	8 (13.3)	
TIMI 3	0 (0.0)	0 (0.0)	
Thrombus aspiration, *n* (%)	4 (6.7)	1 (1.7)	0.364
Number of stents *n* (%)	1.2 ± 0.6	1.3 ± 0.7	0.683
Stent diameter (mm)	3.1 ± 0.4	2.9 ± 0.5	0.163
Total DES length (mm)	24.4 ± 5.5	27.0 ± 6.4	0.017
Post‐TIMI flow grade, *n* (%)			1.000
TIMI 0	0	0	
TIMI 1	0	0	
TIMI 2	1 (1.7)	0	
TIMI 3	59 (98.3)	60 (100.0)	

*Note:* Values are median (IQR) or mean (± standard deviation) and number (percentage).

Abbreviations: C‐PCI, conventional percutaneous coronary intervention; DES, drug‐eluting stent; LAD, left anterior descending artery; LCX, left circumflex artery; LM, left main coronary artery; RCA, right coronary artery; SC‐PCI, single‐catheter percutaneous coronary intervention; TIMI, thrombolysis in myocardial infarction.

### 3.3. Key Procedural Time Metrics

**Figure 2:**
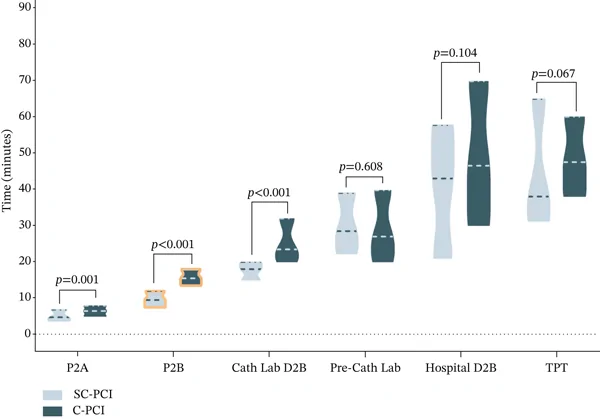
Comparison of key procedural time metrics between SC‐PCI and C‐PCI groups. Abbreviations: P2A, puncture‐to‐angiography; P2B, puncture‐to‐balloon; cath lab D2B, catheterization laboratory door‐to‐balloon; pre‐cath lab, precatheterization laboratory; hospital D2B, hospital door‐to‐balloon; TPT, total procedural time.

For the prespecified primary endpoint, P2B time was significantly shorter in the SC‐PCI group compared with the C‐PCI group (median [IQR]: 9.50 [7.50–12.00] vs. 15.50 [13.50–18.00] min; *p* < 0.001). Consistent with the primary endpoint, SC‐PCI was associated with efficiency advantages across other cath lab metrics: P2A time was significantly shorter (5.00 [4.00–7.00] vs. 6.50 [5.00–8.00] min; *p* = 0.001), as was cath lab D2B time (18.00 [15.00–20.00] vs. 23.50 [20.00–32.00] min; *p* < 0.001), with a trend toward shorter TPT that approached statistical significance (38.00 [31.50–48.00] vs. 42.00 [35.00–55.00] min; *p* = 0.067). Hospital D2B time showed a nonsignificant reduction with SC‐PCI (43.0 [21.0–57.8] vs. 46.5 [30.0–69.8] min; *p* = 0.104), and similar pre‐cath lab times between groups (28.5 [22.3–39.0] vs. 27.0 [20.0–39.7] min; *p* = 0.608) (Figure 2).

### 3.4. Interventional Outcome Measures

Procedural success was comparable between the two groups, achieved in 96.7% of patients in the SC‐PCI group and 100.0% in the C‐PCI group (*p* = 0.496). No device‐related adverse events occurred in the C‐PCI group, and only one event was reported in the SC‐PCI group. The SC‐PCI cohort had significantly shorter fluoroscopy time (median [IQR]: 11.8 [10.4–14.9] vs. 14.3 [11.7–17.6] min; *p* < 0.01) and lower contrast volume (median [IQR]: 120.1 [106.2–140.0] vs. 140.0 [114.7–150.0] mL; *p* = 0.032), whereas total radiation dose was comparable (1839.9 ± 532.3 vs. 1952.7 ± 513.7 mGy; *p* = 0.240). In terms of clinical safety outcomes, the overall complication rates and in‐hospital mortality were numerically similar between the SC‐PCI and C‐PCI groups, with no statistically significant differences (all *p* > 0.05). Two in‐hospital deaths occurred in the SC‐PCI group, both adjudicated as unrelated to the SC‐PCI procedure: One patient with extensive anterior myocardial infarction complicated by cardiogenic shock died on hospital Day 3; the other developed left ventricular free wall rupture on postprocedure Day 2 and died despite resuscitative efforts (Table 3).

**Table 3:** Interventional outcome measures between SC‐PCI and C‐PCI groups.

Variables	SC‐PCI (*n* = 60)	C‐PCI (*n* = 60)	*p*
Procedure success rate, *n* (%)	58 (96.7)	60 (100.0)	0.496
Device safety, *n* (%)	59 (98.3)	60 (100.0)	1.000
Fluoroscopy time (min)	11.8 (10.4–14.9)	14.25 (11.7–17.6)	< 0.01
Contrast volume (mL)	120.1 (106.2–140.0)	140.0 (114.7–150.0)	0.032
Radiation dose (Gy)	1839.9 ± 532.3	1952.7 ± 513.7	0.240
Complications, *n* (%)	6 (10.0)	7 (11.7)	0.769
Length of hospital stay (days)	7.0 (5.0–10.0)	8 (6–10)	0.230

*Note:* Values are median (IQR) or mean (± standard deviation) and number (percentage).

Abbreviations: C‐PCI, conventional percutaneous coronary intervention; SC‐PCI, single‐catheter percutaneous coronary intervention.

### 3.5. Subgroup Analyses

**Figure 3:**
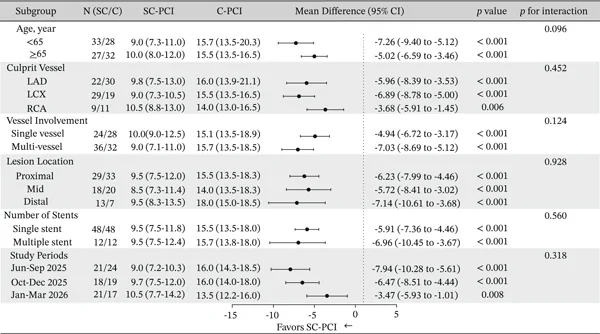
Exploratory subgroup analysis of P2B time between SC‐PCI and C‐PCI groups. *Note:* Data for SC‐PCI and C‐PCI groups are presented as median (interquartile range [IQR]) of P2B time (minutes). *p* values for within‐subgroup comparisons were derived from Mann–Whitney *U* tests. Mean differences (MD) with 95% confidence intervals (CI) and *p* values for interaction were derived from linear regression models. N (SC/C) indicates the number of patients in each subgroup. Study periods were defined as follows: June–September 2025, October–December 2025, and January–March 2026. Abbreviations: SC‐PCI, single‐catheter percutaneous coronary intervention; C‐PCI, conventional two‐catheter percutaneous coronary intervention; P2B, puncture‐to‐balloon; CI, confidence interval; LAD, left anterior descending artery; LCX, left circumflex artery; RCA, right coronary artery; MD, mean difference.

Prespecified exploratory subgroup analyses were performed to evaluate the beneficial effect of SC‐PCI on reducing P2B time across clinical and angiographic subsets, including age, culprit vessel, extent of vessel involvement, lesion location, number of implanted stents, and study periods. Consistent with the overall cohort findings, SC‐PCI was associated with a significantly shorter P2B time compared with C‐PCI in all tested subgroups (all *p* < 0.05). Notably, no significant treatment‐by‐subgroup interaction was observed for any of the examined variables (all *p* for interaction > 0.05) (Figure 3).

## 4. Discussion

The core priority of emergency STEMI management lies in minimizing myocardial ischemia time to achieve rapid reperfusion of the infarct‐related artery, thereby salvaging jeopardized myocardium and improving long‐term prognosis [12, 13]. Extensive evidence confirms that each 30‐min reduction in total ischemia time correlates with a 7.5% lower relative 1‐year mortality, whereas each 1‐min reperfusion delay in the early phase causes irreversible necrosis of millions cardiomyocytes, directly impairing postprocedural cardiac function and long‐term quality of life [12, 14, 15].

Against this background, the core advantage of SC‐PCI is improved cath lab procedural efficiency. Using P2B time—an endpoint spanning radial artery puncture to first balloon inflation—as our primary metric, we observed a significant reduction in this parameter in the SC‐PCI group versus C‐PCI. Unlike conventional endpoints such as D2B or symptom‐to‐balloon (S2B) time, P2B excludes prehospital and patient‐related confounders, enabling more precise assessment of cath lab procedural efficiency, as it isolates the impact of workflow optimization from nonprocedural delays such as in‐hospital administrative preparation [3, 16, 17]. Consistently, we also observed synchronized reductions in P2A and cath lab D2B time, verifying the full‐process optimization of the single‐catheter strategy, rather than isolated speedup of a single step. This efficiency improvement can be attributed to two synergistic mechanisms: First, SC‐PCI eliminates the redundant step of guiding catheter exchange post diagnostic angiography in traditional workflows, enabling seamless transition between angiographic assessment and intervention [16]. Second, our center has implemented SC‐PCI as a routine standardized pathway for emergency STEMI care, with specialized operator training and a cross‐team integrated response protocol, which amplifies the strategy′s inherent efficiency advantage. Notably, the 6‐min reduction in P2B time did not translate into a statistically significant difference in hospital D2B time. This may be attributable to pre‐cath lab procedures, including emergency department evaluation, patient transfer, and informed consent, which attenuate the relative improvement derived from intraprocedural workflow optimization. Additionally, the 3.5‐min numerical difference in D2B may represent a real clinical effect that did not reach significance owing to the limited sample size. Nonetheless, even modest procedural time savings may confer cumulative clinical benefits.

Beyond the observed improved intraprocedural efficiency, SC‐PCI was associated with lower intraprocedural burden in this cohort, including shorter fluoroscopy time and lower contrast medium volume. Reduced contrast volume may potentially lower the risk of contrast‐induced acute kidney injury (CI‐AKI), an established independent predictor of poor prognosis in STEMI [18, 19]. As prior studies including the HORIZONS‐AMI trial have demonstrated that CI‐AKI is associated with significantly higher long‐term all‐cause mortality [20]. This potential benefit may be particularly relevant for high‐risk STEMI patients with comorbidities such as renal insufficiency or diabetes, who are more vulnerable to contrast‐related renal injury [21]. Additionally, shorter fluoroscopy time may translate into lower cumulative radiation exposure for both interventional operators and patients, which has implications for occupational health and long‐term radiation‐related risks [22–24]. However, our study was not powered for clinical endpoints such as CI‐AKI or radiation‐related adverse events, and this potential benefit requires confirmation in larger studies.

No statistically significant differences were observed in procedural success, postprocedural TIMI 3 flow, or complication rates between groups, suggesting comparable procedural safety profiles in this cohort. Of the two in‐hospital deaths in the SC‐PCI group, both were adjudicated as unrelated to the procedure: one from refractory cardiogenic shock complicating and one from left ventricular free wall rupture. Additionally, we observed that total implanted DES length was significantly shorter in the SC‐PCI group. We speculate this is associated with earlier reperfusion: earlier infarct‐related artery opening reduced myocardial edema, allowing operators to accurately assess true lesion extent under near‐physiologic conditions, avoiding overstenting [25]. This aligns with prior reports that earlier reperfusion reduces edema‐driven overestimation of lesion length [26].

Prior studies, limited by sample size and design, mostly relied on post hoc exploratory analyses with methodological limitations including insufficient power and multiple testing bias, leading to uncertain subgroup conclusions [4, 27]. Some small‐sample studies even reported attenuated SC‐PCI benefits in complex lesions, reinforcing clinical concerns that restricted the strategy to only simple lesions [6, 7]. Our exploratory subgroup analysis evaluated consistency of the P2B time benefit across multiple angiographic strata, including culprit vessel, lesion complexity, lesion location, and number of implanted stents. The direction of benefit was across all examined subgroups, with no statistically significant treatment‐by‐subgroup interactions. These findings suggest that the P2B benefit of SC‐PCI may extend to more complex presentations including multivessel disease or proximal coronary lesions. This provides preliminary support for broad applicability of the strategy in STEMI care, which could be particularly valuable in the time‐sensitive setting of primary PCI by simplifying preprocedural decision‐making.

## 5. Limitations

Our study has several limitations. First, this was a single‐center, retrospective, unblinded, nonrandomized study. The primary endpoint of P2B time is operator‐dependent, so performance bias and selection bias cannot be excluded. Although PSM was used to balance confounders, residual confounding remains, and causal inferences should be made with caution. Second, limited by sample size, the study was underpowered for safety endpoints, and all subgroup analyses were exploratory only; larger studies are needed to definitively characterize the safety profile of SC‐PCI. Third, the generalizability of our findings is limited, as the study exclusively used the IL universal guiding catheter and only enrolled patients via transradial access. Finally, only short‐term in‐hospital clinical outcomes were assessed, with no long‐term follow‐up data.

## 6. Conclusion

In conclusion, among STEMI patients undergoing transradial pPCI, SC‐PCI was associated with shorter P2B time and improved intraprocedural efficiency, with no significant differences in periprocedural safety outcomes. These findings suggest that SC‐PCI represents a promising strategy to streamline STEMI interventional workflows and provide a foundation for future multicenter, randomized validation of this approach.

**Nomenclature**
Cath lab: catheterization laboratory
CI: confidence interval
CI‐AKI: contrast‐induced acute kidney injury
C‐PCI: conventional two‐catheter percutaneous coronary intervention
D2B: door‐to‐balloon time
DES: drug‐eluting stent
IL: Ikari Left guiding catheter
IQR: interquartile range
IRA: infarct‐related artery
LAD: left anterior descending coronary artery
LCX: left circumflex coronary artery
MD: mean difference
P2A: puncture‐to‐angiography time
P2B: puncture‐to‐balloon time
pPCI: primary percutaneous coronary intervention
PSM: propensity score matching
RCA: right coronary artery
SC‐PCI: single‐catheter percutaneous coronary intervention
STEMI: ST‐segment elevation myocardial infarction
TIMI: thrombolysis in myocardial infarction
TPT: total procedural time

## Author Contributions

Xin Kou and Sen Lei contributed equally as cofirst authors.

## Funding

No funding was received for this manuscript.

## Disclosure

The authors have nothing to report.

## Ethics Statement

The study protocol was approved by the Institutional Review Board (IRB) of The First Affiliated Hospital of Chongqing Medical University, with a waiver of written informed consent granted due to the retrospective, noninterventional design.

## Conflicts of Interest

The authors declare no conflicts of interest.

## Supporting Information

Additional supporting information can be found online in the Supporting Information section.

## Supporting information


**Supporting Information 1** Appendix S1: Endpoint definitions.

**Supporting Information 2** STROBE statement.

## Data Availability

The datasets analyzed during the current study are available from the corresponding author on reasonable request.
