# Supplementary material for: Single‐Catheter Versus Conventional Two‐Catheter Primary PCI in STEMI: A 1:1 Propensity Score‐Matched Cohort Study
Source: Cardiovasc Ther. 2026 Aug 22;2026:4057540. doi: 10.1155/cdr/4057540 (PMC13498964; doi:10.1155/cdr/4057540)
Supplement: Supplementary file 1 [file CDR-2026-4057540-s001.docx]

STROBE Statement—checklist of items that should be included in reports of observational studies

|  | Item No. | Recommendation | Page  No. | Relevant text from manuscript |
| --- | --- | --- | --- | --- |
| **Title and abstract** | 1 | (*a*) Indicate the study’s design with a commonly used term in the title or the abstract | 1 | Title clearly states 1:1 Propensity Score-Matched Cohort Study |
|  |  | (*b*) Provide in the abstract an informative and balanced summary of what was done and what was found | 3-4 | Abstract summarizes study design, grouping, primary endpoint (P2B time), secondary endpoints, key findings, and conclusions |
| Introduction | | | |  |
| Background/rationale | 2 | Explain the scientific background and rationale for the investigation being reported | 5-6 | STEMI requires urgent reperfusion; pPCI is gold-standard; conventional two-catheter workflow causes delays, higher radiation/contrast exposure, and vascular complications; single-catheter pPCI optimizes workflow but high-quality evidence is limited |
| Objectives | 3 | State specific objectives, including any prespecified hypotheses | 6 | To compare efficacy and safety of SC-PCI vs. C-PCI in STEMI; evaluate procedural time, radiation/contrast exposure, procedural success, safety, and short-term outcomes; prespecified subgroup analyses to verify consistency |
| Methods | | | |  |
| Study design | 4 | Present key elements of study design early in the paper | 6 | Single-center, retrospective observational cohort study; 1:1 propensity score matching (PSM) |
| Setting | 5 | Describe the setting, locations, and relevant dates, including periods of recruitment, exposure, follow-up, and data collection | 6 | Single institutional cardiac catheterization laboratory; study period: June 1, 2025 – March 31, 2026; retrospective data collection; in-hospital short-term follow-up |
| Participants | 6 | (*a*) *Cohort study*—Give the eligibility criteria, and the sources and methods of selection of participants. Describe methods of follow-up  *Case-control study*—Give the eligibility criteria, and the sources and methods of case ascertainment and control selection. Give the rationale for the choice of cases and controls  *Cross-sectional study*—Give the eligibility criteria, and the sources and methods of selection of participants | 6 | (a) Inclusion: STEMI patients undergoing transradial pPCI; Exclusion: age <18 years, cardiogenic shock, mechanical circulatory support, temporary pacemaker, maintenance hemodialysis, incomplete data; in-hospital follow-up |
|  |  | (*b*) *Cohort study*—For matched studies, give matching criteria and number of exposed and unexposed  *Case-control study*—For matched studies, give matching criteria and the number of controls per case | 6 | (b) 1:1 PSM; final matched cohort: 60 SC-PCI, 60 C-PCI |
| Variables | 7 | Clearly define all outcomes, exposures, predictors, potential confounders, and effect modifiers. Give diagnostic criteria, if applicable | 8-9 | Exposure: SC-PCI vs. C-PCI strategy; Outcomes: P2B time, procedural metrics, radiation/contrast dose, complications, in-hospital mortality; Confounders: age, sex, BMI, hypertension, diabetes, renal function, LVEF, Killip class |
| Data sources/ measurement | 8* | For each variable of interest, give sources of data and details of methods of assessment (measurement). Describe comparability of assessment methods if there is more than one group | 6 | Data source: institutional cardiac catheterization laboratory database; Measurements: lab tests, coronary angiography, procedural records, radiation dosimetry, contrast volume; identical assessment methods in both groups |
| Bias | 9 | Describe any efforts to address potential sources of bias | 8 | 1:1 PSM to balance baseline confounders; standardized operator experience, devices, antiplatelet/anticoagulation protocols to reduce performance bias |
| Study size | 10 | Explain how the study size was arrived at | 6 | Consecutive enrollment of eligible patients; 268 patients after exclusion; 1:1 PSM yielded 120 patients (60 per group) |

Continued on next page

| Quantitative variables | 11 | Explain how quantitative variables were handled in the analyses. If applicable, describe which groupings were chosen and why | 9 | Normal distribution: mean ± SD; non-normal: median (IQR) |
| --- | --- | --- | --- | --- |
| Statistical methods | 12 | (*a*) Describe all statistical methods, including those used to control for confounding | 9 | Student’s t-test/Mann–Whitney U test, χ²/Fisher’s exact test; PSM for confounding adjustment |
|  |  | (*b*) Describe any methods used to examine subgroups and interactions | 9-10 | Prespecified 5 subgroup analyses; multivariable linear regression for treatment–subgroup interaction |
|  |  | (*c*) Explain how missing data were addressed | 6 | Patients with incomplete data excluded |
|  |  | (*d*) *Cohort study*—If applicable, explain how loss to follow-up was addressed  *Case-control study*—If applicable, explain how matching of cases and controls was addressed  *Cross-sectional study*—If applicable, describe analytical methods taking account of sampling strategy | 9 | No loss to follow-up (in-hospital short-term study) |
|  |  | (*e*) Describe any sensitivity analyses | 9 | No sensitivity analysis performed |
| Results | | | | |
| Participants | 13* | (a) Report numbers of individuals at each stage of study—eg numbers potentially eligible, examined for eligibility, confirmed eligible, included in the study, completing follow-up, and analysed | Fig.1 | 304 initially screened; 36 excluded; 268 eligible; 120 after 1:1 PSM |
|  |  | (b) Give reasons for non-participation at each stage | Fig.1 | Exclusions: age, cardiogenic shock, temporary pacemaker, hemodialysis, incomplete data, other ineligibility |
|  |  | (c) Consider use of a flow diagram | Fig.1 | Study flow diagram provided (Fig.1) |
| Descriptive data | 14* | (a) Give characteristics of study participants (eg demographic, clinical, social) and information on exposures and potential confounders | Table1; Table2 | (a) Baseline clinical and angiographic characteristics fully reported (Table1, Table2) |
|  |  | (b) Indicate number of participants with missing data for each variable of interest |  | (b) No missing data for key variables |
|  |  | (c) *Cohort study*—Summarise follow-up time (eg, average and total amount) | 6 | (c) In-hospital follow-up only; no long-term follow-up duration |
| Outcome data | 15* | *Cohort study*—Report numbers of outcome events or summary measures over time | Table3; Fig.2 | P2B time, fluoroscopy time, contrast volume, complications, in-hospital mortality, procedural success reported for both groups |
|  |  | *Case-control study—*Report numbers in each exposure category, or summary measures of exposure |  |  |
|  |  | *Cross-sectional study—*Report numbers of outcome events or summary measures |  |  |
| Main results | 16 | (*a*) Give unadjusted estimates and, if applicable, confounder-adjusted estimates and their precision (eg, 95% confidence interval). Make clear which confounders were adjusted for and why they were included | Table1 | PSM-adjusted between-group comparisons; median (IQR) and P-values reported; all baseline confounders adjusted |
|  |  | (*b*) Report category boundaries when continuous variables were categorized | Table3 | Age cutoff at 65 years; clear boundaries for all categorized variables |
|  |  | (*c*) If relevant, consider translating estimates of relative risk into absolute risk for a meaningful time period |  | Absolute risk translation not performed |

Continued on next page

| Other analyses | 17 | Report other analyses done—eg analyses of subgroups and interactions, and sensitivity analyses | Fig.3 | Prespecified 5 subgroup analyses (age, culprit vessel, vessel involvement, lesion location, stent number); no significant treatment–subgroup interaction; consistent benefit of SC-PCI |
| --- | --- | --- | --- | --- |
| Discussion | | | | |
| Key results | 18 | Summarise key results with reference to study objectives | 10 | SC-PCI significantly reduced P2B, P2A, and Cath Lab D2B time; lowered fluoroscopy time and contrast volume; procedural success and safety comparable to C-PCI |
| Limitations | 19 | Discuss limitations of the study, taking into account sources of potential bias or imprecision. Discuss both direction and magnitude of any potential bias | 16 | Single-center retrospective design; residual confounding possible; operators had specialized SC-PCI training; only short-term in-hospital outcomes; transradial access only |
| Interpretation | 20 | Give a cautious overall interpretation of results considering objectives, limitations, multiplicity of analyses, results from similar studies, and other relevant evidence | 16-17 | SC-PCI optimizes STEMI catheter laboratory workflow, accelerates reperfusion, reduces intraprocedural exposure, with non-inferior safety; suitable for routine clinical use |
| Generalisability | 21 | Discuss the generalisability (external validity) of the study results | 14-15-16 | Subgroup analyses confirm consistent benefit across age, coronary anatomy, and lesion complexity; requires multicenter and long-term validation |
| Other information | |  | | |
| Funding | 22 | Give the source of funding and the role of the funders for the present study and, if applicable, for the original study on which the present article is based |  | No specific funding was received for this study |

*Give information separately for cases and controls in case-control studies and, if applicable, for exposed and unexposed groups in cohort and cross-sectional studies.

**Note:** An Explanation and Elaboration article discusses each checklist item and gives methodological background and published examples of transparent reporting. The STROBE checklist is best used in conjunction with this article (freely available on the Web sites of PLoS Medicine at http://www.plosmedicine.org/, Annals of Internal Medicine at http://www.annals.org/, and Epidemiology at http://www.epidem.com/). Information on the STROBE Initiative is available at www.strobe-statement.org.
