# Supplementary material for: Single‐Catheter Versus Conventional Two‐Catheter Primary PCI in STEMI: A 1:1 Propensity Score‐Matched Cohort Study
Source: Cardiovasc Ther. 2026 Aug 22;2026:4057540. doi: 10.1155/cdr/4057540 (PMC13498964; doi:10.1155/cdr/4057540)
Supplement: Supplementary file 2 [file CDR-2026-4057540-s002.docx]

**Single-Catheter Versus Conventional Two-Catheter Primary PCI in STEMI: A 1:1 Propensity Score-Matched Cohort Study**

**Appendix 1. Endpoint Definitions**

**Primary Endpoint**

Puncture-to-balloon (P2B) time: Time interval from successful radial arterial puncture and sheath insertion to the first balloon dilatation of the culprit infarct-related artery

**Secondary Endpoints**

**1. Core Procedural Time Metrics**

- Puncture-to-angiography (P2A) time: Interval from successful radial arterial puncture and sheath insertion to acquisition of the first diagnostic coronary angiogram, (min)
- Cath lab door-to-balloon (D2B) time: Interval from patient arrival at the cath lab entrance to first balloon dilatation of the culprit artery
- Pre-cath lab time: Interval from hospital emergency department arrival to cath lab entry, (min)
- Hospital D2B time: Interval from patient arrival at the hospital emergency department entrance to first balloon dilatation of the culprit artery, (min)
- Total procedural time (TPT): Interval from arterial puncture to final sheath removal at the conclusion of the procedure, (min)

**2. Intraprocedural Exposure Metrics**

- Fluoroscopy time: Total duration of fluoroscopic imaging during the entire procedure, (min)
- Contrast volume: Total volume of iodinated contrast medium administered during the procedure, (mL)
- Total radiation dose: Cumulative patient radiation exposure during the procedure, (mGy)

**3. Procedural Performance and Device Safety Metrics**

- Procedure success rate: Defined as successful completion of PCI with final TIMI grade 3 flow in the culprit artery and residual stenosis <10% after stenting
- Device safety rate: Defined as the proportion of patients free of intraprocedural device-related adverse events, including catheter fracture, catheter-induced dissection, balloon rupture, stent malapposition, and device embolization

**4. Clinical Safety and Short-term Outcome Metrics**

- Overall complication rate: Incidence of any adverse event related to the interventional procedure during index hospitalization
- Length of hospital stay: Total number of days from hospital admission to discharge
- In-hospital mortality: Death from any cause occurring during index hospitalization
